# Supplementary material for: Morphometric differentiation of three chicken ecotypes of Ethiopia using multivariate analysis
Source: PLoS One. 2024 Feb 28;19(2):e0295134. doi: 10.1371/journal.pone.0295134 (PMC10901329; doi:10.1371/journal.pone.0295134)
Supplement: S1 Table — (DOCX) [file pone.0295134.s001.docx]

**S1 Table 1**. Mean, minimum, and maximum values for quantitative traits of the three female chicken ecotypes

| Traits | Lowland | | | Midland | | | Highland | | |
| --- | --- | --- | --- | --- | --- | --- | --- | --- | --- |
|  | Mean | Min | Max | Mean | Min | Max | Mean | Min | Max |
| Body length (cm) | 28.23 | 26.35 | 30.50 | 24.92 | 22.72 | 27.72 | 23.53 | 21.53 | 25.03 |
| Body weight(g) | 1320 | 1304 | 1436 | 1270 | 1030 | 1382 | 1191.7 | 956.60 | 1297.50 |
| Shank length(cm) | 9.814 | 8.50 | 10.20 | 8.289 | 7.850 | 8.90 | 7.270 | 6.40 | 7.850 |
| Comb length (cm) | 3.235 | 3.1 | 4.0 | 2.067 | 1.20 | 3.30 | 2.781 | 2.20 | 3.70 |
| Comb width (cm) | 1.451 | 1.150 | 1.60 | 1.756 | 1.60 | 2.00 | 1.223 | 0.950 | 1.50 |
| Comb index | 2.247 | 2.047 | 3.389 | 1.1779 | 0.6176 | 1.7632 | 2.285 | 1.731 | 3.083 |
| Earlobe length(cm) | 1.626 | 1.55 | 1.70 | 1.271 | 0.950 | 1.50 | 1.884 | 1.700 | 2.50 |
| Earlobe width(cm) | 0.9128 | 0.80 | 1.10 | 0.7057 | 0.50 | 0.900 | 1.225 | 0.800 | 1.600 |
| Earlobe index | 1.836 | 1.750 | 2.333 | 1.840 | 1.250 | 2.60 | 1.566 | 1.188 | 2.444 |
| Wattle length(cm) | 2.501 | 2.30 | 3.70 | 1.613 | 0.90 | 3.750 | 2.076 | 1.00 | 2.60 |
| Wattle width(cm) | 1.377 | 1.00 | 2.150 | 1.0761 | 0.750 | 2.100 | 1.392 | 0.650 | 2.20 |
| Wattle index | 1.922 | 1.633 | 5.478 | 1.56 | 1.230 | 3.288 | 1.507 | 1.103 | 1.959 |
| Skull length(cm) | 3.950 | 5.783 | 6.00 | 6.274 | 4 | 7.900 | 6.382 | 3.60 | 7.00 |
| Skull width(cm) | 3.405 | 2.250 | 3.950 | 3.514 | 1.850 | 4.100 | 3.585 | 2.100 | 4.75 |
| Skull index | 1.756 | 1.413 | 2.90 | 1.821 | 1.604 | 2.663 | 1.805 | 1.151 | 2.762 |
| Neck length(cm) | 13.93 | 13.00 | 15.75 | 15.61 | 13.00 | 26.50 | 12.15 | 10.45 | 13.50 |
| Beak length(cm) | 2.033 | 1.65 | 2.25 | 2.065 | 1.200 | 2.65 | 2.168 | 1.80 | 4.00 |
| Beak width (cm) | 1.076 | 0.70 | 1.550 | 0.9267 | 0.560 | 1.400 | 1.034 | 0.850 | 1.350 |
| Beak index | 1.965 | 1.576 | 4.500 | 2.351 | 1.875 | 4.610 | 2.128 | 1.583 | 4.737 |
| Spur length(cm) | 0.5116 | 0.350 | 0.70 | 0.2729 | 0.125 | 0.550 | 0.4119 | 0.350 | 0.500 |
| Wing span (cm) | 40.13 | 35.60 | 45.60 | 34.39 | 29.80 | 37.70 | 32.83 | 28.00 | 36.50 |
