# Supplementary material for: Morphometric differentiation of three chicken ecotypes of Ethiopia using multivariate analysis
Source: PLoS One. 2024 Feb 28;19(2):e0295134. doi: 10.1371/journal.pone.0295134 (PMC10901329; doi:10.1371/journal.pone.0295134)
Supplement: S2 Table — (DOCX) [file pone.0295134.s002.docx]

**S2 Table 2.** Mean, minimum, and maximum values for quantitative traits of the three male chicken ecotypes

| Traits | Lowland | | | Midland | | | Highland | | |
| --- | --- | --- | --- | --- | --- | --- | --- | --- | --- |
|  | Mean | Min | Max | Mean | Min | Max | Mean | Min | Max |
| Body length (cm) | 39.53 | 35.50 | 42.25 | 36.08 | 35.02 | 38.72 | 32.50 | 26.70 | 35.50 |
| Body weight(g) | 1676 | 1390 | 1928 | 1579 | 1458 | 1699 | 1459 | 1364 | 1528 |
| Shank length(cm) | 12.93 | 10.00 | 14.50 | 10.81 | 9.95 | 12.50 | 9.865 | 7.965 | 10.865 |
| Comb length (cm) | 7.499 | 6.40 | 8.50 | 6.676 | 4.950 | 7.850 | 5.467 | 2.40 | 6.40 |
| Comb width (cm) | 3.815 | 2.950 | 4.60 | 2.754 | 1.950 | 3.400 | 3.086 | 2.650 | 3.635 |
| Comb index | 1.979 | 1.623 | 2.469 | 2.440 | 1.768 | 3.083 | 1.7785 | 0.7273 | 2.2545 |
| Earlobe length(cm) | 3.554 | 3.00 | 4.250 | 2.528 | 2.00 | 2.800 | 3.056 | 2.30 | 3.400 |
| Earlobe width(cm) | 2.186 | 1.750 | 2.850 | 1.321 | 0.950 | 1.900 | 1.859 | 1.450 | 2.450 |
| Earlobe index | 1.642 | 1.329 | 2.167 | 1.961 | 1.333 | 2.842 | 1.658 | 1.224 | 2.138 |
| Wattle length(cm) | 6.208 | 4.900 | 7.700 | 4.456 | 3.400 | 5.600 | 5.299 | 4.70 | 6.200 |
| Wattle width(cm) | 3.934 | 2.800 | 5.950 | 2.773 | 1.900 | 3.600 | 3.371 | 2.750 | 4.30 |
| Wattle index | 1.611 | 1.224 | 2.203 | 1.707 | 1.250 | 3.077 | 1.665 | 1.069 | 2.256 |
| Skull length(cm) | 6.626 | 4.10 | 8.400 | 7.044 | 4.80 | 8..90 | 6.629 | 3.500 | 8.20 |
| Skull width(cm) | 4.177 | 1.620 | 6.565 | 4.180 | 2.30 | 5.750 | 3.873 | 2.950 | 5.200 |
| Skull index | 1.637 | 2.212 | 2.872 | 1.718 | 1.227 | 2.209 | 1.734 | 1.082 | 2.441 |
| Neck length(cm) | 15.70 | 14.50 | 17.50 | 16.93 | 15.00 | 19.54 | 13.70 | 12.25 | 15.50 |
| Beak length(cm) | 2.234 | 2.100 | 2.450 | 2.074 | 1.850 | 2.30 | 1.988 | 1.650 | 2.208 |
| Beak width (cm) | 1.244 | 1.050 | 1.450 | 0.9708 | 0.6800 | 1.2450 | 1.039 | 0.865 | 1.230 |
| Beak index | 1.803 | 1.500 | 2.182 | 2.174 | 1.625 | 2.933 | 1.931 | 1.545 | 2.254 |
| Spur length(cm) | 2.444 | 1.950 | 3.00 | 1.482 | 0.950 | 1.900 | 1.869 | 1.250 | 2.400 |
| Wing span (cm) | 47.52 | 40.50 | 52.00 | 40.01 | 34.40 | 44.90 | 36.80 | 28.600 | 40.600 |
