# Supplementary material for: Morphometric differentiation of three chicken ecotypes of Ethiopia using multivariate analysis
Source: PLoS One. 2024 Feb 28;19(2):e0295134. doi: 10.1371/journal.pone.0295134 (PMC10901329; doi:10.1371/journal.pone.0295134)
Supplement: Attachment — Submitted filename: Authors response .doc [file pone.0295134.s003.doc]

# Authors’ responses to Reviewer (PONE-S-23-16017)

Manuscript Number: **PONE-S-23-16017**

## Manuscript title: Morphometric Differentiation of Three Chicken Ecotypes of Ethiopia Using Multivariate Analysis

**Response to Editors:** we authors greatly appreciated all the inputs from reviewers, which have been much contributed in improving our manuscript’s quality. Here under we gave point-by- point response to comments of the reviewer and incorporated in the revision version of the manuscript.

Response to Reviewer 1:

Thanks indeed for the constructive comment and we have revised the comments in the manuscript and incorporated in the revised version of the manuscript as suggested

## :

**Comment 1:** What is the standard to use almost 1:1 ratio of male and female (146 male and 164 females) chicken in your study

Answer: We used the FAO (2012) guideline: the morphometric characterization requires at least 10–30 cocks and 100–300 hens. **As sample size increases, precision improves**. Based on this concept, a purposive random sampling technique was used to select 770 indigenous chickens (412 females and 358 males): 310 lowland chickens (146 males and 164 females), 260 midland chickens (120 males and 140 females), and 200 highland chickens (92 males and 108 females)

**Comment 2:** you already have fixed the chickens in to their perspective agro-ecologies; so, why you need to conduct cluster analysis?

Answer: All farmers of the three agro ecologies used the market in the lowland agroecology for indigenous chicken products (live chicken and eggs). This is mainly due to:

- Indigenous chicken products from both highland and midland are always collected and transported either by local chicken producers and/or local chicken traders to the lowland agroecology of the zone during every occasion, especially Ethiopian religious festivals and the Ethiopian New Year, as the price of chicken products in both agro ecologies (midland and highland) is much cheaper than in the lowland.
- There is a seasonal movement of farmers and their domestic animals, particularly oxen, donkeys, and chickens, from both agro-ecologies to lowland agro-ecologies every rainy season to cultivate either their own or rented land as there is a scarcity of cultivated land in both agro-ecologies (midland and highland).

Because of the above reasons, there is a probability of mixing among the three indigenous chicken ecotypes.

**Comment 3:** Some inconsistencies…. Please use either” local” or “indigenous” for the chicken ecotypes you used for the study. My recommendation is to say “indigenous” rather than saying “local” along the manuscript.

**Answer:** we replaced local with indigenous along the manuscript as suggested.

**Responses to reviewer 2:**

**Comment 1**: Introduction: Ethiopia is thought to have the largest livestock population…………………million beehives. The reference for that data is of 2016. Please include the data from recent references

**Answer:** we replaced the old reference by recent reference: CSA (2021) as commented.

**Comment 2:** Poor understanding of the production system and absence of comprehensive breeding strategies Efforts to boost the performance……..’ write the sentence properly

**Answer: we wrote the sentences like:** This might be attributed to poor nutrition, the presence of diseases and predators, institutional and socioeconomic constraints, limited management skills, a poor understanding of the production system, and the absence of comprehensive breeding strategies [**3-8**]. as commented.

**Comment 3:** ‘Baseline information on…………………….utilization and improvements’. Split the sentence and make simple sentences for easy understanding.

**Answer**: We split the sentence like**:** Baseline information on well-adapted indigenous chicken gene pool diversity is very crucial for the conservation of chicken genetic resources and maintaining the genetic variations within and among them to fulfill present and future market demands**.** **It serves** as insurance against environmental changes, such as changes in production circumstances on a socio-economic, historic, and cultural level, and to provide adequate genetic sources for ensuring sustainable utilization and improvements.

**Comment 4:** Split the sentence and make simple sentences for easy understanding.

Answer: we split the sentence like**:** Rosario *et al*. [17] also reported that the mechanisms involved in the control of morphological traits in chickens are too complex to be explained only under univariate analysis**.** **All associated** traits are biologically correlated due to Pleiotropic effect of genes and loci linkages.

**Comment 5**: Multivariate statistical techniques are suitable approaches to analyzing……’ replace ‘**to’** with **‘for’**

**Answer:** we replaced ‘**to’** with **‘for’** as suggested**.**

**Comment 6:** In this study only some morphometric traits were measured so how the genetic diversity was estimated in the study?

Answer: We corrected the statement with: Thus, this study was designed to differentiate the three indigenous chicken ecotypes of the western zone of the Tigray regional state of Ethiopia by using multivariate analysis and considering morphometric traits

**Comment 7:** The materials and methods section needs more detail. This is especially important in the light of strong efforts on the part of the scientific community to improve replicability

Answer: We made the materials and methods more detail: by incorporating sampling technique after description of the study area, and we also incorporated the statistical model used under the data analysis.

**Comment 8:** The Results section needs clarification and tighter organization.

Answer: we improved the clarification and organization of the result section as commented.

**Comment 9:** The discussion section needs to be tightened up with respect to writing. It is overly long and quite speculative.
**Answer:** We improved the discussion section as suggested

**Comment 10**: Conclusion section is also lengthy reduce the section and keep only the salient findings.

Answer: We reduced the conclusion section as commented.

## Responses to editor’s comments:

**Comment 1:** Please ensure that your manuscript meets PLOS ONE's style requirements, including those for file naming. The PLOS ONE style templates can be found at

Answer: We ensured our manuscript meets PLOS ONE’s style requirements by referring the PLOS ONE style templates.

**Comment 2:** We note that the grant information you provided in the ‘Funding Information’ and ‘Financial Disclosure’ sections do not match.

When you resubmit, please ensure that you provide the correct grant numbers for the awards you received for your study in the ‘Funding Information’ section.

**Answer:** We made the Funding Information and financial Disclosure sections similar as suggested.

**Comment 3:** Thank you for stating the following in the Acknowledgments Section of your manuscript: We note that you have provided funding information that is not currently declared in your Funding Statement. However, funding information should not appear in the Acknowledgments section or other areas of your manuscript. We will only publish funding information present in the Funding Statement section of the online submission form. Please remove any funding-related text from the manuscript and let us know how you would like to update your Funding Statement.

**Answer:** We removed the funding statement from the manuscript (acknowledgments section) as commented.

**Comment 4:** In your Data Availability statement, you have not specified where the minimal data set underlying the results described in your manuscript can be found. PLOS defines a study's minimal data set as the underlying data used to reach the conclusions drawn in the manuscript and any additional data required to replicate the reported study findings in their entirety. All PLOS journals require that the minimal data set be made fully available.

**Answer:** We incorporated the minimal data as suggested

**Comment 5:** PLOS requires an ORCID iD for the corresponding author in Editorial Manager on papers submitted after December 6th, 2016. Please ensure that you have an ORCID iD and that it is validated in Editorial Manager. To do this, go to ‘Update my Information’ (in the upper left-hand corner of the main menu), and click on the Fetch/Validate link next to the ORCID field. This will take you to the ORCID site and allow you to create a new iD or authenticate a pre-existing iD in Editorial Manager. Please see the following video for instructions on linking an ORCID iD to your Editorial Manager account

**Answer:** The corresponding author created an ORCID iD**: 00000-0002-4820-1237**

**Comment 6:** Please include your full ethics statement in the ‘Methods’ section of your manuscript file. In your statement, please include the full name of the IRB or ethics committee who approved or waived your study, as well as whether or not you obtained informed written or verbal consent. If consent was waived for your study, please include this information in your statement as well.

**Answer:** we included the ethical statement in the methods section before the description of the study area as commented**.**

**Comment 7:** We note that Figure 1] in your submission contain [map/satellite] images which may be copyrighted. All PLOS content is published under the Creative Commons Attribution License (CC BY 4.0), which means that the manuscript, images, and Supporting Information files will be freely available online, and any third party is permitted to access, download, copy, distribute, and use these materials in any way, even commercially, with proper attribution. For these reasons, we cannot publish previously copyrighted maps or satellite images created using proprietary data, such as Google software (Google Maps, Street View, and Earth).

Answer: we removed Figure 1 from the manuscript totally as suggested.

**Comment 8:** 8. Please review your reference list to ensure that it is complete and correct. If you have cited papers that have been retract, please include the rationale for doing so in the manuscript text, or remove these references and replace them with relevant current references. Any changes to the reference list should be mentioned in the rebuttal letter that accompanies your revised manuscript. If you need to cite a retracted article, indicate the article’s retracted status in the References list and also include a citation and full reference for the retraction notice.
**Answer:** We checked the reference is complete and correct as suggested. We replaced some old references with recent references.
