# Supplementary material for: Morphometric differentiation of three chicken ecotypes of Ethiopia using multivariate analysis
Source: PLoS One. 2024 Feb 28;19(2):e0295134. doi: 10.1371/journal.pone.0295134 (PMC10901329; doi:10.1371/journal.pone.0295134)
Supplement: Attachment — Submitted filename: Authors response .doc [file pone.0295134.s004.doc]

# Authors’ responses to Reviewer (PONE-S-23-16017)

Manuscript Number: **PONE-S-23-16017**

## Manuscript title: Morphometric Differentiation of Three Chicken Ecotypes of Ethiopia Using Multivariate Analysis

**Response to Editors:** we authors greatly appreciated all the inputs from reviewers, which have been much contributed in improving our manuscript’s quality. Here under we gave point-by- point response to comments of the reviewer and incorporated in the revision version of the manuscript.

## Responses to editor’s comments:

Thanks indeed for the constructive comment and we have revised the comments in the manuscript and incorporated in the revised version of the manuscript as suggested

**Comment 1:** Need a linguistic reformulation of the English language I recommend sending it to the authorities editing language

Answer: We edited and improved the English language of the whole portion of the manuscript (starting from Abstract up to conclusion) as suggested.

**Comment 2:** The difference of ages affects the measured data, so the effect of age must be included in the statistical model used to analyze the data.

Answer: We fellow a general guideline for the age ranges that can be considered for minimizing the effect of age on individual **body measurements:**

1. **Body weight:** Chickens generally reach their maximum body weight and stop growing at around **18-22** weeks of age, depending on the breed. After this age, their growth rate slows down significantly, and they may continue to gain weight slowly over time due to factors like diet and exercise.
2. Wingspan: Wingspan measurements can be taken when the chickens are around 12 to 16 weeks old. At this age, the wings have reached a significant proportion of their adult size.
3. Shank length: The shanks, which refer to the lower leg and foot of the chicken, usually reach their full length and size by this time. Chickens typically stop shank development by around 16-20 weeks of age. However, it's important to note that individual chickens may vary in their growth patterns, so some may continue to have slight growth or changes in their shanks beyond this age.
4. Comb width and length, skull width and length, earlobe width and length, wattle width and length: These measurements are often taken when the chickens are around 20 to 24 weeks old. By this age, the comb, skull, earlobes, and wattles have usually reached their adult size and shape.
5. Spur length: Spur length measurements are typically conducted in adult chickens, which are usually around 30 to 36 weeks old. This is when the spurs have fully developed.
6. Beak width and length: Beak measurements can be taken when the chickens are around 8 to 12 weeks old. At this age, the beak has generally reached its adult size.

In fact, we took all layers from each of the three female chicken ecotypes whose ages range from 10 month up to 12 months, as well as one cock with 6-month-old, one cock with 9 -month-old and four cocks with 11-month-old from the highland male chicken ecotype. The ages of the remaining 86 cocks of the highland male chicken ecotype, as well as the midland (120cocks) and lowland (146 cocks) chicken ecotypes are 12 month.

**Responses to reviewer 1:**

**Comment 1**: I have satisfied; but for another time while working such kind of works, it is better to balance the number of male and female animals following the guideline for phenotypic characterization of animals

**Answer:** Thank you very much for your valuable and constructive comments.

**Responses to reviewer 2:**

**Comment 1:** English language needs to be thoroughly edited and improved. Use of unscientific words and unnecessarily long sentences has to be avoided and the overall content needs to be made more concise and clear

Answer: We edited and improved the English language of the whole portion of the manuscript (starting from the abstract up to the Conclusion), as well as making the whole portion concise and clear as suggested.

Methods:

**Comment 2:** Sampling technique: 6 months old or older chicken seems to be vague criteria especially in the light of the fact that inclusion of older chickens may influence the individual trait measurements and may hide the true picture of the traits in the three ecotypes. Age should be considered as a separate factor for more accuracy in results

**Answer:** We fellow a general guideline for the age ranges that can be considered for minimizing the effect of age on individual body measurements:

7. **Body weight:** Chickens generally reach their maximum body weight and stop growing at around **18-22** weeks of age, depending on the breed. After this age, their growth rate slows down significantly, and they may continue to gain weight slowly over time due to factors like diet and exercise.
8. Wingspan: Wingspan measurements can be taken when the chickens are around 12 to 16 weeks old. At this age, the wings have reached a significant proportion of their adult size.
9. Shank length: The shanks, which refer to the lower leg and foot of the chicken, usually reach their full length and size by this time. Chickens typically stop shank development by around 16-20 weeks of age. However, it's important to note that individual chickens may vary in their growth patterns, so some may continue to have slight growth or changes in their shanks beyond this age.
10. Comb width and length, skull width and length, earlobe width and length, wattle width and length: These measurements are often taken when the chickens are around 20 to 24 weeks old. By this age, the comb, skull, earlobes, and wattles have usually reached their adult size and shape.
11. Spur length: Spur length measurements are typically conducted in adult chickens, which are usually around 30 to 36 weeks old. This is when the spurs have fully developed.
12. Beak width and length: Beak measurements can be taken when the chickens are around 8 to 12 weeks old. At this age, the beak has generally reached its adult size.

In fact, we took all layers from each of the three female chicken ecotypes whose ages range from 10 month up to 12 months, as well as one cock with 6-month-old, one cock with 9 -month-old and four cocks with 11-month-old from the highland male chicken ecotype. The ages of the remaining 86 cocks of the highland male chicken ecotype, as well as the midland (120cocks) and lowland (146 cocks) chicken ecotypes are 12 month.

**Comment 3:** Selection of farmers based on possession of 3 or more chickens: Three numbers of chickens is too less to provide the accurate description of morphometric traits and hence, characterize a population

**Answer:** Farmers possessing three or more chickens refers to farmers, who have at least two breeding hens and one breeding cock, or more, excluding chicks, pullets, and cockerels. Based on the chicken production potential of the area, we believe this is sufficient for characterizing the chicken population in the area.

**Comment 4:** Please describe the visible appearance of the chicken ecotypes

**Answer:** All female and male chicken ecotypes have normal feather morphology and distribution.

Even though the frequency of plumage color distribution varied among female and male chicken ecotypes, the observed plumage colors in female chicken ecotypes were as follows: red (1^st^), grayish (2^nd^), multicolored (3^rd^), white with red spots (4^th^), white (5^th^), and black (6^th^). Similarly, in male chicken ecotypes, the detected plumage colors were red (1^st^), multicolored (2^nd^), grayish (3^rd^), white with red spots (4^th^), white (5^th^), and black (6^th^).

**Comment 5:** The same temperature in the study area of both Weynadega (midland) and Dega (highland) seems confusing as different ecological regions should have different temperatures and other weather conditions

**Answer:** We corrected the annual temperature of the agro-ecologies and we rewrite the sentence like: The annual temperature ranges from **27oC to 45^o^C** in Kolla, **15^o^C to 30^o^C** in Weynadega and **10^o^C to 22^o^C** in Dega areas. It was a typing error.

**Comment 6:** Population size for different ecotypes is less so as to warrant enough accuracy

**Answer:** This is based on the chicken production potential of the area; we believe this is sufficient for characterizing the chicken population in the area.

**Results**

**.**

**Comment 7:** PCA and step wise discriminant analysis provided enough clarity regarding the discrimination of the populations and the most important traits for discriminating the three populations. Was there some special reason as to **why Canonical discriminant analysis was done?**

Answer: The use of Canonical Discriminant Analysis (CDA) in addition to PCA and stepwise discriminant analysis may have been done for several reasons:

1. **Enhanced discrimination**: CDA is specifically designed to maximize the separation between groups or populations. The incorporation of CDA may have aimed to further enhance the discrimination between the populations and identify the most significant traits for differentiation.
2. Multivariate analysis: CDA allows for the analysis of multiple variables simultaneously. This can be useful when there are multiple traits or characteristics being considered for discrimination. The inclusion of CDA allows for the assessment of the combined effect of multiple variables on population differentiation.
3. Validation and robustness: CDA can provide additional validation and robustness to the findings obtained from PCA and stepwise discriminant analysis. It can help confirm the results and ensure that the identified traits for discrimination are consistent and reliable.
4. Interpretation of results: CDA can provide interpretable results in terms of canonical discriminant functions. These functions can indicate the relative importance of different variables in discriminating between populations. Including CDA can facilitate a more comprehensive interpretation of the findings.

Overall, the addition of Canonical Discriminant Analysis to the analysis framework may have been done to further enhance the discrimination between populations, validate the results, and provide additional insights into the most important traits for differentiation.

**Comment 8:** 7 PCs for discriminating female chicken ecotypes does not seems to make much sense…Comment

**Answer:** The following are the possible reasons for the difference in the number of principal components for discriminating female and male chicken ecotypes in the area.

- **Genetic differences**: Female and male chickens may exhibit different genetic variations, leading to distinct principal components that contribute to their discrimination. These genetic differences could be related to traits such as body size, feather coloration, or reproductive characteristics.
- **Phenotypic variation**: Female and male chickens may display different phenotypic characteristics that contribute to the principal components. For example, males may have more prominent combs or different feather patterns compared to females, resulting in distinct principal components for their discrimination.
- **Sexual dimorphism:** Sexual dimorphism, which refers to physical differences between males and females of the same species, could contribute to the variation in principal components. Traits that are specific to one sex, such as wattles or tail feathers in males, may contribute to the discrimination between male chicken ecotypes.
- **Behavioral differences:** Male and female chickens may exhibit different behaviors, such as mating displays or territorial behaviors, which could contribute to the variation in principal components. These behavioral differences may be reflected in the data used for the principal component analysis.

**Responses to reviewer 3:**

**Comment 1:** English language needs to be thoroughly edited and improved. Use of unscientific words and unnecessarily long sentences has to be avoided and the overall content needs to be made more concise and clear

Answer: We edited and improved the English language of the whole portion of the manuscript (starting from the abstract up to the Conclusion), as well as making the whole portion concise and clear as suggested.

Methods:

**Comment 2:** Sampling technique: 6 months old or older chicken seems to be vague criteria especially in the light of the fact that inclusion of older chickens may influence the individual trait measurements and may hide the true picture of the traits in the three ecotypes. Age should be considered as a separate factor for more accuracy in results

**Answer:** We fellow a general guideline for the age ranges that can be considered for minimizing the effect of age on individual body measurements:

13. **Body weight:** Chickens generally reach their maximum body weight and stop growing at around **18-22** weeks of age, depending on the breed. After this age, their growth rate slows down significantly, and they may continue to gain weight slowly over time due to factors like diet and exercise.
14. Wingspan: Wingspan measurements can be taken when the chickens are around 12 to 16 weeks old. At this age, the wings have reached a significant proportion of their adult size.
15. Shank length: The shanks, which refer to the lower leg and foot of the chicken, usually reach their full length and size by this time. Chickens typically stop shank development by around 16-20 weeks of age. However, it's important to note that individual chickens may vary in their growth patterns, so some may continue to have slight growth or changes in their shanks beyond this age.
16. Comb width and length, skull width and length, earlobe width and length, wattle width and length: These measurements are often taken when the chickens are around 20 to 24 weeks old. By this age, the comb, skull, earlobes, and wattles have usually reached their adult size and shape.
17. Spur length: Spur length measurements are typically conducted in adult chickens, which are usually around 30 to 36 weeks old. This is when the spurs have fully developed.
18. Beak width and length: Beak measurements can be taken when the chickens are around 8 to 12 weeks old. At this age, the beak has generally reached its adult size.

In fact, we took all layers from each of the three female chicken ecotypes whose ages range from 10 month up to 12 months, as well as one cock with 6-month-old, one cock with 9 -month-old and four cocks with 11-month-old from the highland male chicken ecotype. The ages of the remaining 86 cocks of the highland male chicken ecotype, as well as the midland (120cocks) and lowland (146 cocks) chicken ecotypes are 12 month.

**Comment 3:** Selection of farmers based on possession of 3 or more chickens: Three numbers of chickens is too less to provide the accurate description of morphometric traits and hence, characterize a population

**Answer:** Farmers possessing three or more chickens refers to farmers, who have at least two breeding hens and one breeding cock, or more, excluding chicks, pullets, and cockerels. Based on the chicken production potential of the area, we believe this is sufficient for characterizing the chicken population in the area.

**Comment 4:** Please describe the visible appearance of the chicken ecotypes

**Answer:** All female and male chicken ecotypes have normal feather morphology and distribution.

Even though the frequency of plumage color distribution varied among female and male chicken ecotypes, the observed plumage colors in female chicken ecotypes were as follows: red (1^st^), grayish (2^nd^), multicolored (3^rd^), white with red spots (4^th^), white (5^th^), and black (6^th^). Similarly, in male chicken ecotypes, the detected plumage colors were red (1^st^), multicolored (2^nd^), grayish (3^rd^), white with red spots (4^th^), white (5^th^), and black (6^th^).

**Comment 5:** The same temperature in the study area of both Weynadega (midland) and Dega (highland) seems confusing as different ecological regions should have different temperatures and other weather conditions

**Answer:** We corrected the annual temperature of the agro-ecologies and we rewrite the sentence like: The annual temperature ranges from **27oC to 45^o^C** in Kolla, **15^o^C to 30^o^C** in Weynadega and **10^o^C to 22^o^C** in Dega areas. It was a typing error.

**Comment 6:** Population size for different ecotypes is less so as to warrant enough accuracy

**Answer:** This is based on the chicken production potential of the area; we believe this is sufficient for characterizing the chicken population in the area.

**Results**

**.**

**Comment 7:** PCA and step wise discriminant analysis provided enough clarity regarding the discrimination of the populations and the most important traits for discriminating the three populations. Was there some special reason as to **why Canonical discriminant analysis was done?**

Answer: The use of Canonical Discriminant Analysis (CDA) in addition to PCA and stepwise discriminant analysis may have been done for several reasons:

5. **Enhanced discrimination**: CDA is specifically designed to maximize the separation between groups or populations. The incorporation of CDA may have aimed to further enhance the discrimination between the populations and identify the most significant traits for differentiation.
6. Multivariate analysis: CDA allows for the analysis of multiple variables simultaneously. This can be useful when there are multiple traits or characteristics being considered for discrimination. The inclusion of CDA allows for the assessment of the combined effect of multiple variables on population differentiation.
7. Validation and robustness: CDA can provide additional validation and robustness to the findings obtained from PCA and stepwise discriminant analysis. It can help confirm the results and ensure that the identified traits for discrimination are consistent and reliable.
8. Interpretation of results: CDA can provide interpretable results in terms of canonical discriminant functions. These functions can indicate the relative importance of different variables in discriminating between populations. Including CDA can facilitate a more comprehensive interpretation of the findings.

Overall, the addition of Canonical Discriminant Analysis to the analysis framework may have been done to further enhance the discrimination between populations, validate the results, and provide additional insights into the most important traits for differentiation.

**Comment 8:** 7 PCs for discriminating female chicken ecotypes does not seems to make much sense…Comment

**Answer:** The following are the possible reasons for the difference in the number of principal components for discriminating female and male chicken ecotypes in the area.

- **Genetic differences**: Female and male chickens may exhibit different genetic variations, leading to distinct principal components that contribute to their discrimination. These genetic differences could be related to traits such as body size, feather coloration, or reproductive characteristics.
- **Phenotypic variation**: Female and male chickens may display different phenotypic characteristics that contribute to the principal components. For example, males may have more prominent combs or different feather patterns compared to females, resulting in distinct principal components for their discrimination.
- **Sexual dimorphism:** Sexual dimorphism, which refers to physical differences between males and females of the same species, could contribute to the variation in principal components. Traits that are specific to one sex, such as wattles or tail feathers in males, may contribute to the discrimination between male chicken ecotypes.
- **Behavioral differences:** Male and female chickens may exhibit different behaviors, such as mating displays or territorial behaviors, which could contribute to the variation in principal components. These behavioral differences may be reflected in the data used for the principal component analysis.
