# Supplementary material for: Morphometric differentiation of three chicken ecotypes of Ethiopia using multivariate analysis
Source: PLoS One. 2024 Feb 28;19(2):e0295134. doi: 10.1371/journal.pone.0295134 (PMC10901329; doi:10.1371/journal.pone.0295134)
Supplement: Attachment — Submitted filename: comments R2.docx [file pone.0295134.s005.docx]

The article entitled” Morphometric **Differentiation of Three Chicken Ecotypes of Ethiopia Using Multivariate Analysis**” has scientific merit and useful information. The authors have addressed the queries raised by me during the Review process. The article may be accepted for publication in PLOS one

(U.Rajkumar)

Principal Scientist & Head

Poultry Genetics & Breeding
