# Supplementary material for: Morphometric differentiation of three chicken ecotypes of Ethiopia using multivariate analysis
Source: PLoS One. 2024 Feb 28;19(2):e0295134. doi: 10.1371/journal.pone.0295134 (PMC10901329; doi:10.1371/journal.pone.0295134)
Supplement: Attachment — Submitted filename: Authors response .doc [file pone.0295134.s006.doc]

# Authors’ responses to Reviewer (PONE-D-23-12190R2) [EMID: 5ba6b263e0608c79]

Manuscript Number: **PONE-D-23-12190R2**

## Manuscript title: Morphometric Differentiation of Three Chicken Ecotypes of Ethiopia Using Multivariate Analysis

**Response to Editors:** we authors greatly appreciated all the inputs from reviewers, which have been much contributed in improving our manuscript’s quality. Here under we gave point-by- point response to comments of the reviewer and incorporated in the revision version of the manuscript.

## Responses to editor’s comments:

Thanks indeed for the constructive comment and we have revised the comments in the manuscript and incorporated in the revised version of the manuscript as suggested

**Comment 1:** The conclusion section needs to be refined before publication because there are many repeats with the results section.

Answer: We edited and refined the conclusion section by avoiding repetition with the result section. Additionally, we reduced its size from 166 words to 153 words as suggested.

**Comment 2:** English language of this paper needs checking.

Answer: The English language of the entire manuscript portion was checked by senior animal breeding scientists, namely the second author, Professor Berhanu Belay (PhD), and the third author, Dr. Tadelle Dessie (PhD), as suggested.

**Responses to reviewer 1:**

**Comment 1**: The article entitled” Morphometric Differentiation of Three Chicken Ecotypes of Ethiopia Using Multivariate Analysis” has scientific merit and useful information. The authors have addressed the queries raised by me during the Review process. The article may be accepted for publication in PLOS one

**Answer:** Thank you very much for forwarding your valuable comments to support the acceptance of our manuscript for publication.

**Responses to reviewer 2:**

**Comment 1:** The conclusion section need to be refined before publication, because there are many repeats with the results section

Answer: We edited and refined the conclusion section by avoiding repetition with the result section. Additionally, we reduced its size from 166 words to 153 words as suggested.

**Responses to reviewer 3:**

**Comment 1**: The article entitled” Morphometric Differentiation of Three Chicken Ecotypes of Ethiopia Using Multivariate Analysis” has scientific merit and useful information. The authors have addressed the queries raised by me during the Review process. The article may be accepted for publication in PLOS one

**Answer:** Thank you very much for forwarding your valuable comments to support the acceptance of our manuscript for publication.

**Responses to reviewer 4:**

**Comment 1:** The conclusion section need to be refined before publication, because there are many repeats with the results section.

Answer: We edited and refined the conclusion section by avoiding repetition with the result section. Additionally, we reduced its size from 166 words to 153 words as suggested.
