# Supplementary material for: Morphometric differentiation of three chicken ecotypes of Ethiopia using multivariate analysis
Source: PLoS One. 2024 Feb 28;19(2):e0295134. doi: 10.1371/journal.pone.0295134 (PMC10901329; doi:10.1371/journal.pone.0295134)
Supplement: Attachment — Submitted filename: Authors response .doc [file pone.0295134.s007.doc]

# Authors’ responses to Reviewer (PONE-D-23-12190R3) [EMID: 5ba6b263e0608c79]

Manuscript Number: **PONE-D-23-12190R3**

## Manuscript title: Morphometric Differentiation of Three Chicken Ecotypes of Ethiopia Using Multivariate Analysis

**Response to Editors:** we authors greatly appreciated all the inputs from editors/reviewers, which have been much contributed in improving our manuscript’s quality. Here under we gave point-by- point response to comments of the editor/reviewer and incorporated in the revision version of the manuscript.

## Responses to editor’s comments:

Thanks indeed for the constructive comment and we have revised the comments in the manuscript and incorporated in the revised version of the manuscript as suggested

**Comment 1:**  During our final internal checks on this submission, we noticed that this manuscript is very closely related to the following papers, of which you are an author:

Morpho-biometric characterization of indigenous chicken ecotypes in North-western Ethiopia", published in PLOS ONE (<https://doi.org/10.1371/journal.pone.0286299>).

1.1. We ask you make references pone.0286299 in your manuscript and contextualize the study relates to this previous work.

Answer: We incorporated and cited this article (**PONE.0286299) in** our manuscript**’s references and we also** contextualized our study relative this previous work as suggested.

1.2. Please clarify the differences in the research questions addressed and the data sets used in these related manuscripts

##  Answer: The currently done research, "Morphometric Differentiation of Three Chicken Ecotypes of Ethiopia (western zone of Tigray regional state of Ethiopia) Using Multivariate Analysis" (PONE-D-23-12190), and the previously done research, "Morpho-biometric characterization of indigenous chicken ecotypes in North-western Ethiopia (in three zones of Amhara regional state of Ethiopia)" published in PLOS ONE (PONE.0286299), differ in their research questions and data sets.

## Firstly, the current study specifically aims to differentiate between three indigenous chicken ecotypes in the western zone of the Tigray regional state of Ethiopia using multivariate analysis. This represents a targeted investigation into a specific region and a specific differentiation goal. In contrast, the previous study focused on the morpho-biometric characterization of indigenous chicken ecotypes in the Amhara regional state, encompassing three zones. This earlier research had a broader scope, focusing on characterizing ecotypes across a larger geographic area.

## Secondly, the data sets used in these studies are distinct. The current study employs data specific to the western zone of the Tigray regional state, while the previous research used data from the three zones of the Amhara regional state. This difference in data sets reflects the specific geographical and ecological contexts of the respective studies.

## Thirdly, the current study area (western zone of Tigray) is geographically isolated from the study area of the published paper by physical barriers: Tekeze river, *Angreb* river (*Bahras-salam* river) and the highest Volcanic mountain in Ethiopia namely *Rasdashan (RasDashen*/ *Ras Dejen*) and other smaller mountains like *Tikldngay* mountain. In addition to this, the current study Area is located 694 .6 kilometers far from the study areas (three zones of Amhara regional state of Ethiopia) of the published paper.

## In summary, the current study is more narrowly focused on differentiating specific chicken ecotypes in the western zone of the Tigray regional state using multivariate analysis, while the previous research had a broader scope, characterizing ecotypes across multiple zones in the Amhara regional state. Additionally, the data sets used in each study are tailored to the specific geographic regions under investigation.”

Generally, the indigenous chicken population studied under the current study (western zone of Tigray) and the indigenous chicken populations of the three zones of Amhara regional state of Ethiopia for published paper is completely two different chicken populations because they **are isolated by physical barriers and they are geographically isolated by 694.6 kilometers**. Researches on the isolated chicken populations of different areas of the same country are conducted for the following reasons:

**Genetic drift:** Isolation can lead to genetic drift, where random processes cause the gene pool of a population to change over time. This can result in differences in genetic makeup between the isolated populations, leading to distinct indigenous chicken populations**.**

**Founder effect:** If a small group of chickens initially colonized one area and a different group colonized the other, the genetic diversity of the founding populations may have been different. Over time, this can lead to divergence in the genetic composition of the two populations.

**Adaptation to local conditions:** Even within the same agro-ecological zone, there may be microclimatic and ecological differences between the isolated areas. Over time, indigenous chicken populations may adapt to these local conditions, leading to differences in traits such as foraging behavior, disease resistance, and physical characteristics**.**

**Human influence:** Cultural and management practices, including selective breeding, may differ between the isolated areas. This can lead to different phenotypic traits being favored in each population, contributing to morphological differences.

**Historical factors:** Historical events, such as human migrations or changes in land use, may have influenced the distribution and genetic makeup of indigenous chicken populations in the isolated areas.

**Environmental barriers:** Physical barriers can affect the movement and exchange of chicken populations, limiting gene flow between areas and allowing genetic differences to accumulate.

Overall, the combination of genetic, environmental, and human factors can lead to the divergence of indigenous chicken populations in isolated areas within the same country or agro-ecological zone.

1.3. Explain how the work described in your PLOS ONE submission advances on that described in this related paper

Answer:

The current work, "Morphometric Differentiation of Three Chicken Ecotypes of Ethiopia (western zone of Tigray regional state of Ethiopia) Using Multivariate Analysis" (PONE-D-23-12190), advances on the previously done research, "Morpho-biometric characterization of indigenous chicken ecotypes in North-western Ethiopia (in three zones of Amhara regional state of Ethiopia)" published in PLOS ONE (PONE.0286299), in the following ways.

**Firstly,** the current work focuses on a specific region, the western zone of the Tigray regional state of Ethiopia, while the previous research examined three zones of the Amhara regional state. This more targeted approach allows for a more in-depth investigation into the morphometric differentiation of chicken ecotypes in this specific region.

**Secondly**, the current work employs a multivariate analysis to differentiate between three indigenous chicken ecotypes, while the previous research used morpho-biometric characterization. Multivariate analysis is a more advanced statistical technique that allows for a more comprehensive analysis of multiple variables, which can provide a more nuanced understanding of differences between the ecotypes.

**Thirdly,** the current work studies larger quantitative traits than the previous research, with a total of twenty-one morphometric traits of the three ecotypes. These larger quantitative traits allow for a more comprehensive examination of the morphological traits and can potentially reveal unique features and differences between the chicken ecotypes.

Overall, the current work represents a more targeted and advanced approach to investigating the morphometric differentiation of indigenous chicken ecotypes in Ethiopia, specifically in the western zone of the Tigray regional state. The use of multivariate analysis of twenty- one morphometric traits can provide a more comprehensive understanding of the differences between the ecotypes in this region.”

**Responses to for all reviewers:**

**Comment 1**: The article entitled” Morphometric Differentiation of Three Chicken Ecotypes of Ethiopia Using Multivariate Analysis” has scientific merit and useful information. The authors have addressed the queries raised by me during the Review process. The article should be accepted for publication in PLOS one

**Answer:**  Thank you very much for forwarding your valuable comments to support the acceptance of our manuscript for publication.
