# Supplementary material for: Morphometric differentiation of three chicken ecotypes of Ethiopia using multivariate analysis
Source: PLoS One. 2024 Feb 28;19(2):e0295134. doi: 10.1371/journal.pone.0295134 (PMC10901329; doi:10.1371/journal.pone.0295134)
Supplement: Attachment — Submitted filename: Authors response .doc [file pone.0295134.s008.doc]

Authors’ responses to Reviewer (**PONE-D-23-12190R4**) [EMID: 5ba6b263e0608c79]

Manuscript Number: **PONE-D-23-12190R4**

## Manuscript title: Morphometric Differentiation of Three Chicken Ecotypes of Ethiopia Using Multivariate Analysis

**Response to Editors:** we authors greatly appreciated all the inputs from editors/reviewers, which have been much contributed in improving our manuscript’s quality. Here under we gave point-by- point response to comments of the editor/reviewer and incorporated in the revision version of the manuscript.

## Responses to editor’s comments:

Thanks indeed for the constructive comment and we have revised the comments in the manuscript and incorporated in the revised version of the manuscript as suggested

**Comment 1:**  Thank you to the author for the response to clarify the differences between the previous and current manuscript, but your responses are only for the auditors. This was **not clear** in the **introduction** and **discussed results** of the manuscript. Please clarify the differences between the **previous** and **current research** in the text of writing the manuscript, Please write different **color marks highlighted** in the paragraph and all that is added in this is learned on the highlighter to facilitate its review.

**Answer: I. We** have clarified the differences between the previous and our current research in the **final** section of the **manuscript's introduction**, as suggested below.

“Multiple studies have examined the morphological diversity of various indigenous chicken populations in different areas of Ethiopia using multivariate analysis of morphometric traits [20-25]. However, the indigenous chicken populations prevailing in the western zone of Tigray are entirely distinct from the previously studied indigenous chicken populations. This distinction arises from their isolation by physical barriers, differences in agroecology, and significant geographical distance. Furthermore, there has been no research on differentiating these unique indigenous chicken ecotypes found only in the western zone of Tigray regional state of Ethiopia using multivariate analysis of morphometric traits. This study, therefore, aims to bridge this gap by using multivariate analysis of morphometric traits to differentiate between the three indigenous chicken ecotypes in the western zone of the Tigray regional state of Ethiopia.”

**Answer II**: We have also clarified the differences between the previous and current research in the manuscript's discussion, as indicated below.

- In the first section of the discussion, i.e. at the end of the quantitative traits, we have clarified the difference as indicated below: “The variations in quantitative traits among local chicken populations in different areas of Ethiopia may be attributed to genetic variations, rearing environments, isolation by physical barriers, agroecology, significant geographical distances, human selection pressures, breeding programs, and genetic interactions within the populations.”

In the principal component analysis section of the discussion. We have clarified the differences as shown below:-“The variation in the number of principal components extracted and their explained variance can result from the differences in the genetic makeup of the ecotypes, environmental factors, or measurement methods. The variation in principal components distinguishing female and male chicken ecotypes in the area may be due to genetic differences linked to body size, reproductive traits, phenotypic variation, sexual dimorphism, and behavioral differences like mating displays or territorial behavior.

(<https://statisticsbyjim.com/basics/principal-component-analysis/>.”

## Comment 2: Please review your reference list to ensure that it is complete and correct. If you have cited papers that have been retracted, please include the rationale for doing so in the manuscript text, or remove these references and replace them with relevant current references. Any changes to the reference list should be mentioned in the rebuttal letter that accompanies your revised manuscript. If you need to cite a retracted article, indicate the article’s retracted status in the References list and also include a citation and full reference for the retraction notice.

Answer2: We have reviewed and checked the reference lists, and they are complete and correct. Moreover, we have also checked each reference list, whether retracted or not, using the Retraction Watch Database (<http://retractiondatabase.org/>).  In other words, we searched the Retraction Databases, such as websites like Retraction Watch, which maintain databases of retracted articles. We found that all reference lists of our manuscript do not include retracted papers.

**Comment 3:** While revising your submission, please upload your figure files to the Preflight Analysis and Conversion Engine (PACE) digital diagnostic tool, <https://pacev2.apexcovantage.com/>. PACE helps ensure that figures meet PLOS requirements. To use PACE, you must first register as a user. Registration is free.

**Answer 3:** First, the corresponding author registered as a user of the PACE digital diagnostic tool. Then, we uploaded our figure files to the Preflight Analysis and Conversion Engine (PACE) digital diagnostic tool as suggested. Next, we diagnosed the figures using the tool, and then exported and submitted them as figure files along with the revised version of the manuscript.

**Responses to reviewers:**

Thanks indeed for the constructive comment and we have revised the comments in the manuscript and incorporated in the revised version of the manuscript as suggested

**Comment 1:**  Thank you to the author for the response to clarify the differences between the previous and current manuscript, but your responses are only for the auditors. This was **not clear** in the **introduction** and **discussed results** of the manuscript. Please clarify the differences between the **previous** and **current research** in the text of writing the manuscript, Please write different **color marks highlighted** in the paragraph and all that is added in this is learned on the highlighter to facilitate its review.

**Answer: I. we** have clarified the differences between the previous and our current research in the **final** section of the **manuscript's introduction**, as suggested below.

“Multiple studies have examined the morphological diversity of various indigenous chicken populations in different areas of Ethiopia using multivariate analysis of morphometric traits [20-25]. However, the indigenous chicken populations prevailing in the western zone of Tigray are entirely distinct from the previously studied indigenous chicken populations. This distinction arises from their isolation by physical barriers, differences in agroecology, and significant geographical distance. Furthermore, there has been no research on differentiating these unique indigenous chicken ecotypes found only in the western zone of Tigray regional state of Ethiopia using multivariate analysis of morphometric traits. This study, therefore, aims to bridge this gap by using multivariate analysis of morphometric traits to differentiate between the three indigenous chicken ecotypes in the western zone of the Tigray regional state of Ethiopia.”

**Answer II**: We have also clarified the differences between the previous and current research in the manuscript's discussion, as indicated below.

- In the first section of the discussion, i.e. at the end of the quantitative traits, we have clarified the difference as indicated below: “The variations in quantitative traits among local chicken populations in different areas of Ethiopia may be attributed to genetic variations, rearing environments, isolation by physical barriers, agroecology, significant geographical distances, human selection pressures, breeding programs, and genetic interactions within the populations.”

In the principal component analysis section of the discussion. We have clarified the differences as shown below:-“The variation in the number of principal components extracted and their explained variance can result from the differences in the genetic makeup of the ecotypes, environmental factors, or measurement methods. The variation in principal components distinguishing female and male chicken ecotypes in the area may be due to genetic differences linked to body size, reproductive traits, phenotypic variation, sexual dimorphism, and behavioral differences like mating displays or territorial behavior.

(<https://statisticsbyjim.com/basics/principal-component-analysis/>.”

## Comment 2: Please review your reference list to ensure that it is complete and correct. If you have cited papers that have been retracted, please include the rationale for doing so in the manuscript text, or remove these references and replace them with relevant current references. Any changes to the reference list should be mentioned in the rebuttal letter that accompanies your revised manuscript. If you need to cite a retracted article, indicate the article’s retracted status in the References list and also include a citation and full reference for the retraction notice.

Answer2: We have reviewed and checked the reference lists, and they are complete and correct. Moreover, we have also checked each reference list, whether retracted or not, using the Retraction Watch Database (<http://retractiondatabase.org/>).  In other words, we searched the Retraction Databases, such as websites like Retraction Watch, which maintain databases of retracted articles. We found that all reference lists of our manuscript do not include retracted papers.

**Comment 3:** While revising your submission, please upload your figure files to the Preflight Analysis and Conversion Engine (PACE) digital diagnostic tool, <https://pacev2.apexcovantage.com/>. PACE helps ensure that figures meet PLOS requirements. To use PACE, you must first register as a user. Registration is free.

**Answer 3:** First, the corresponding author registered as a user of the PACE digital diagnostic tool. Then, we uploaded our figure files to the Preflight Analysis and Conversion Engine (PACE) digital diagnostic tool as suggested. Next, we diagnosed the figures using the tool, and then exported and submitted them as figure files along with the revised version of the manuscript.
